# Honokiol induces superoxide production by targeting mitochondrial respiratory chain complex I in *Candida albicans*

**DOI:** 10.1371/journal.pone.0184003

**Published:** 2017-08-30

**Authors:** Lingmei Sun, Kai Liao, Dayong Wang

**Affiliations:** 1 Department of Pharmacology, Medical School of Southeast University, Nanjing, China; 2 Department of Pathology and Pathophysiology, Medical School of Southeast University, Nanjing, China; 3 Key Laboratory of Developmental Genes and Human Disease in Ministry of Education, Medical School of Southeast University, Nanjing, China; Yonsei University, REPUBLIC OF KOREA

## Abstract

**Background:**

Honokiol, a compound extracted from *Magnolia officinalis*, has antifungal activities by inducing mitochondrial dysfunction and triggering apoptosis in *Candida albicans*. However, the mechanism of honokiol-induced oxidative stress is poorly understood. The present investigation was designed to determine the specific mitochondrial reactive oxygen species (ROS)-generation component.

**Methods/results:**

We found that honokiol induced mitochondrial ROS accumulation, mainly superoxide anions (O_2_^•−^) measured by fluorescent staining method. The mitochondrial respiratory chain complex I (C I) inhibitor rotenone completely blocked O_2_^•−^ production and provided the protection from the killing action of honokiol. Moreover, respiratory activity and the C I enzyme activity was significantly reduced after honokiol treatment. The differential gene-expression profile also showed that genes involved in oxidoreductase activity, electron transport, and oxidative phosphorylation were upregulated.

**Conclusions:**

The present work shows that honokiol may bind to mitochondrial respiratory chain C I, leading to mitochondrial dysfunction, accompanied by increased cellular superoxide anion and oxidative stress.

**General significance:**

This work not only provides insights on the mechanism by which honokiol interferes with fungal cell, demonstrating previously unknown effects on mitochondrial physiology, but also raises a note of caution on the use of *M*. *officinalis* as a Chinese medicine due to the toxic for mitochondria and suggests the possibility of using honokiol as chemosensitizer.

## Introduction

Currently, disseminated invasive candidiasis has an approximate estimated mortality rate of 40%, even after being treated with antifungal drugs [1,2]. *Candida albicans* is the major cause of candidiasis and is the fourth most commonly reported nosocomial infection [3–5]. As everyone knows, the majority of living organisms need oxygen to survive. Mitochondrial respiration, dealing with transfer of unpaired electrons to oxygen (O_2_), may produce reactive oxygen species (ROS) such as superoxide anion (O_2_^•−^) and subsequently dismutation of O_2_^•−^ to H_2_O_2_ [6]. Within the mitochondria, the main sites of superoxide production have been localized to the electron transport chain (ETC) complexes I (C I) and Ⅲ [6–9]. The bulk of mitochondrial ROS typically arise because of electron leakage from forward electron transport onto O_2_ during aerobic respiration as side products. It is driven by NADH-linked substrates, C I exhibits only minimal ROS production, but the addition of a ubiquinone-site inhibitor, such as rotenone, results in a significant increase in its rate [7–9]. The other mechanism by which ETC produces large amounts of O_2_^•−^ is during reverse electron transport. During reverse electron transport, driven by succinate, ROS production by C I is increased significantly, and in this case, inhibited by the addition of rotenone [7, 8]. In addition to C I, C Ⅲ is regarded as an important site of O_2_^•−^ production, especially when mitochondrial respiration is suppressed by antimycin, an inhibitor of C Ⅲ[6]. O_2_^•−^ is then dismutated by superoxide dismutases to H_2_O_2_ that is reduced to H_2_O by catalase, peroxiredoxins, and glutathione peroxidases [9].When intracellular levels of ROS are high, ROS can have deleterious effects on cellular biomolecules including protein, lipid, RNA and DNA and cause subsequent cell death [9].

Honokiol, a neolignan isolated from the oriental medicine plant *Magnolia officinalis*, is an interesting compound exhibiting various pharmacological activities in preclinical experimental models [10–14]. Honokiol molecule contains two phenolic groups which can exhibit antioxidant properties similar to vitamin E or polyphenols. A paradox of honokiol is that it may have both pro- and antioxidant activities [15,16]. Previous mechanism studies, including those from our own laboratory, indicated that the apoptosis induction by honokiol in *C*. *albicans* was associated with production of ROS [15]. However, we are still lacking a detailed mechanistic knowledge of the architecture of mitochondrial ROS-producing systems induced by honokiol such as of C I or C Ⅲ and detailed insights on the mechanisms controlling their activities. The present study will make an attempt to clarify specific proposed mitochondrial ROS-producing components after honokiol treatemt.

## Materials and methods

### Materials

Honokiol (5,5’-diallyl-2,4’-dihydroxybiphenyl) was obtained from Xi'an Yuquan Biological Technology Co., Ltd and its purity is over 98% analyzed by high-performance liquid chromatography. DCFH-DA (2′,7′-dichlorofluorescein diacetate), dihydroethidium (DHE), 5-cyano-2,3-ditolyl tetrazolium chloride (CTC), and other molecular grade chemicals were obtained from Sigma Chemicals (St. Louis, MO, U.S.A.).

### Microorganisms

*C*. *albicans* wild type strain SC5314 was used in this study [17]. The strain SC5314 was cultured in YPD (yeast extract/ peptone/dextrose) broth. For agar plates, 2% (w/v) bacto agar (Difco, BD Biosciences) was added to the medium. The strain was stored as frozen stock with 15% (v/v) glycerol at –80◦C. Before each experiment, cells were freshly revived on YPD plate from the stock.

### ROS determination

SC5314 cells were adjusted to 1×10^7^ cells/mL in YPD medium and exposed to different concentration of honokiol at 37°C for 4h. Intracellular ROS concentrations were determined in liquid cultures of *C*. *albicans* after honokiol treatment. H_2_O_2_ and O_2_^•−^ levels were detected by adding DCFH-DA and DHE to the culture, respectively. After staining with 10 μmol/L DCFH-DA or 5 μmol/L DHE at 37°C for 30 min, the cells were collected and washed three times with PBS. The fluorescence intensities of the resuspended cells were measured by a flow cytometer using 488nm excitation and a 515nm band-pass filter for DCF detection and a filter >560nm for DHE detection (Becton-Dickinson Immunocytometry Systems, San Jose, CA).

### Determination of mitochondrial C I activity

Extraction of mitochondrial proteins was performed as previously described [18].The enzyme activity assay of NADH CoQ reductase (mitochondrial C I) was carried out according to the instruction manual of the Mitochondrial C I Assay Kit (Genmed Scientifics, Inc.). Protein quantity was estimated by BCA protein assay kit (Beyotime, China). The C I activities were all normalized by the protein content in each sample and converted to the percentage of the control group.

### Respiratory activity

The tetrazolium salt CTC is frequently used as indicator of microorganisms’ respiration [19]. Reduction of CTC is an indication of respiratory enzyme activity. Respiratory activity was assessed by using CTC (5-cyano-2,3-ditolyl tetrazolium chloride), a monotetrazolium redox dye which produces a CTC-formazan (CTF) fluorescent complex (indicated by cells stained in red) when it is biologically reduced, indicating respiration (metabolic activity). Samples were stained with 2.5mM CTC for 30 min. The respiratory activity was determined by the intensity of fluorescence with flow cytometry. Images of cells were obtained using a fluorescence microscope.

### Viability detection

To perform viability detection, the yeast cells were suspended at a concentration of 10^4^ cells/mL in 5 mL YPD medium with different concentrations of compounds. The tubes were incubated at 37°C without shaking for 24 h, and then 100 μL aliquots were washed, diluted and plated for colony counts on YPD agar medium. The plates were incubated for 48 h at 37°C and colonies were counted to determine CFU per mL.

### RNA isolation, cDNA library construction and sequencing

*C*. *albicans* SC5314 cells were grown overnight at 37°C. The overnight culture was used to inoculate 20 ml of YPD to an initial 1×10^7^ cells/mL, and incubated at 37°C for an additional 4 h at 150 rpm. The cells were then harvested and RNA extraction was performed by using the hot acidic phenol method [20]. Then genomic DNA was removed from RNA sample using DNase (New England Biolabs). RNA purity was assessed using the Nanodrop-2000 (Thermo Scientific, USA). Each RNA sample had an A260:A280 ratio above 1.9 and A260:A230 ratio above 1.8. Total RNA integrity was then subsequently checked using an Agilent Technologies 2100 Bioanalyzer with an RNA Integrity Number (RIN) value greater than or equal to 8. Next, two sequencing libraries were constructed by TruSeq™RNA Sample Preparation Kit according to the product instruction (Illumina). Each library was sequenced using Illumina HiSeq2500 for 2 × 150 bp pair-end (PE) sequencing.

### RNA-Seq data analysis and differentially expressed gene identification

Quality control of all raw reads was conducted by Fastqc (http://www.bioinformatics.babraham.ac.uk/projects/fastqc/) software. An initial filtering step was performed to exclude poor quality reads, including adaptor reads, ambiguous nucleotides and low-quality reads (reads having more than 50% bases with quality value). Then the clean reads were mapped onto the *C*. *albicans* SC5314 reference genome [21] (C_albicans_SC5314_version_A21-s02-m07-r10(http://www.candidagenome.org/download/sequence/C_albicans_SC5314/Assembly21/archive/) independently by TopHat [22] (v2.0.10). The ‘-G’ option of Tophat together with the Gene Transfer Format (GTF) file of the Ensemble gene annotation was used for read mapping. The other parameters were set to default values. Based on the Tophat alignment BAM file, HTSeq [23] (v0.6.1) was used to estimate and quantify gene expression with default parameters, yielding raw read count for each genes. Gene expression was measured in reads per kilobase of exon per million reads mapped (RPKM). Finally, edgeR [24] was used to identify the differentially expressed genes (DEGs) by pairwise comparisons. The difference was considered significant if the FDR ≤0.05.

### Gene ontology (GO) and Kyoto Encyclopedia of Genes and Genomes (KEGG) enrichment analysis

GO enrichment analysis provides all GO terms that are significantly enriched in DEGs, relative to the genome background, and filters the DEGs that correspond to a specific biological functions. This method firstly maps all DEGs to GO terms in the database (http://www.geneontology.org/), calculating gene numbers for every term, and then uses the hyper geometric test to find significantly enriched GO terms in the input list of DEGs. GO: TermFinder (http://smd.stanford.edu/help/GO-TermFinder/GO_TermFinder_help.shtml) was used to enrichment the differentially expressed genes. The calculated p-value goes through Bonferroni Correction, taking corrected p-value ≤0.05 as a threshold.

KEGG pathway enrichment analysis identifies significantly enriched metabolic pathways or signal transduction pathways in the whole genome background using KOBAS [25], taking the FDR≤0.05 as a threshold to identify enrichment pathway.

### Quantitative real-time PCR analysis

Quantitative PCR (qPCR) was performed as described previously [20]. Primer sequences used for the amplification of specific genes are shown in S1 Table.

### Statistical analysis

All data were presented as means ± standard error of the mean (S.E.M.). Graphs were generated using Microsoft Excel (Microsoft Corp., Redmond, WA). Statistical analysis was performed using SPSS 12.0 (SPSS Inc., Chicage, USA). Differences between groups were determined using analysis of variance (ANOVA).A p value < 0.05 was considered significant.

## Results

### Induction of oxidative stress after honokiol treatment on *C*. *albicans*

**Fig 1:**
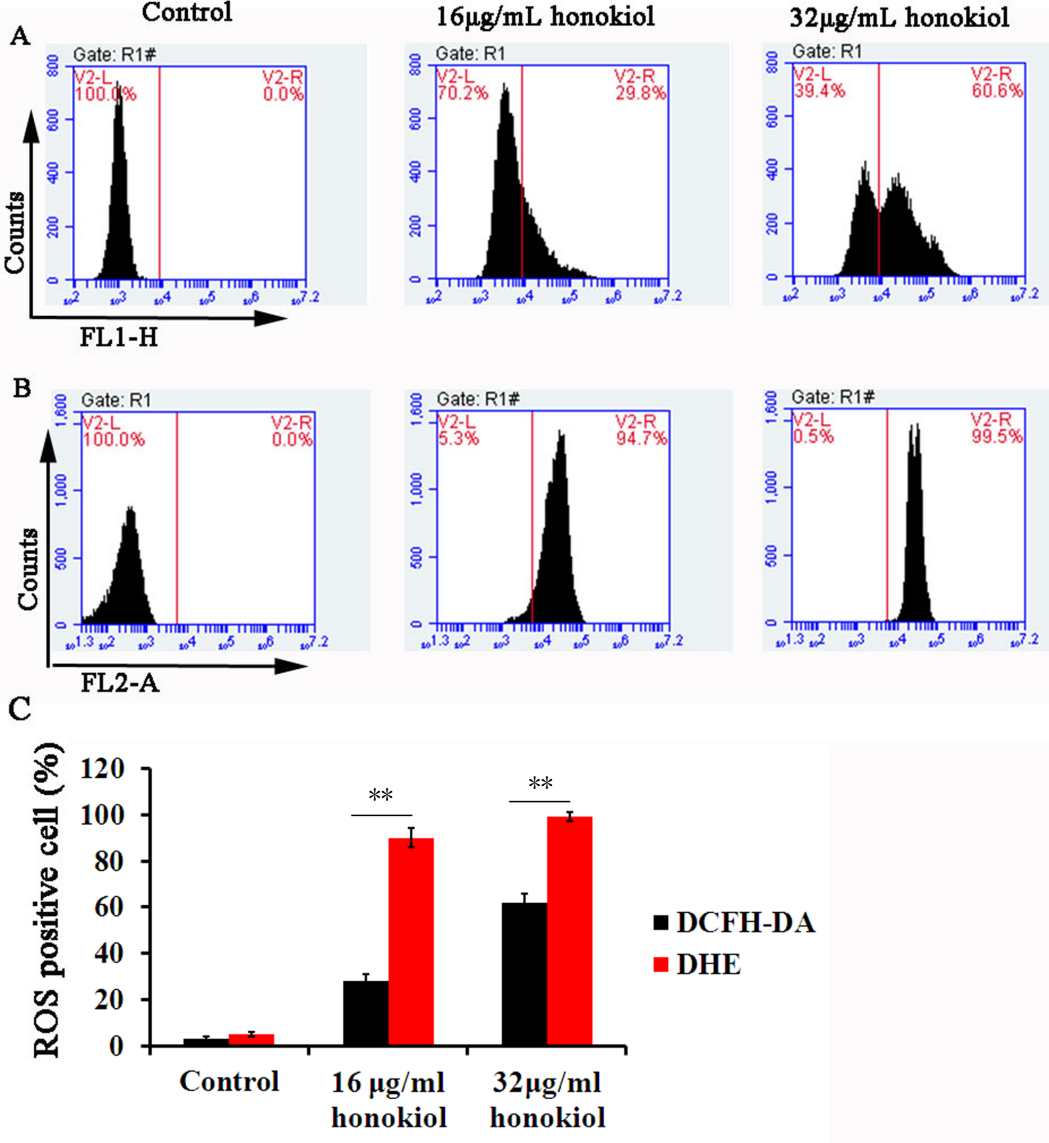
**Levels of H**_**2**_**O**_**2**_ **(A) and O**_**2**_^**•−**^**(B) in *C*.*albicans* SC5314 after honokiol treatment.** (A) and (B) ROS production was evaluated by flow cytometry. (C) The percentage of ROS-positive cells after honokiol treatment. H_2_O_2_ and O_2_^•−^ were determined by fluorometric detection of the oxidation products of DCFH-DA and DHE, respectively. **p<0.01.

The term ROS encompasses oxygen free radicals, such as O_2_^•−^ and H_2_O_2._ The levels of O_2_^•−^ and H_2_O_2_ can be detected by DHE and DCFH-DA staining, respectively [26]. Mitochondria have long been established as a major source of ROS, which is generated from O_2_ by electron leakage or reverse electron transport originating in the mitochondrial transport chain, during respiration [6]. Fig 1A shows that control cells showed little O_2_^•−^ and H_2_O_2_ accumulation, while 16μg/mL honokiol causes an increase in the fluorescence intensity of *C*. *albicans* loaded with DCFH-DA or DHE. The quantification of ROS was performed by flow cytometry. 16μg/mL honokiol induced DCF positive staining in 28.7%±3.1% cells, while most of cells (90.3±3.7%) were DHE positive staining after honokiol exposure. When addition of honokiol at 32μg/mL, the levels of ROS were further increased, 62.0%±3.6% for DCF positive cells and 99.1%±1.8% for DHE positive cells, respectively (Fig 1B). These results concluded that honokiol stimulated ROS formation, mainly O_2_^•−^.

### Effect of respiratory chain inhibitors on the oxidative burst and *C*. *albicans* killing induced by honokiol

**Fig 2:**
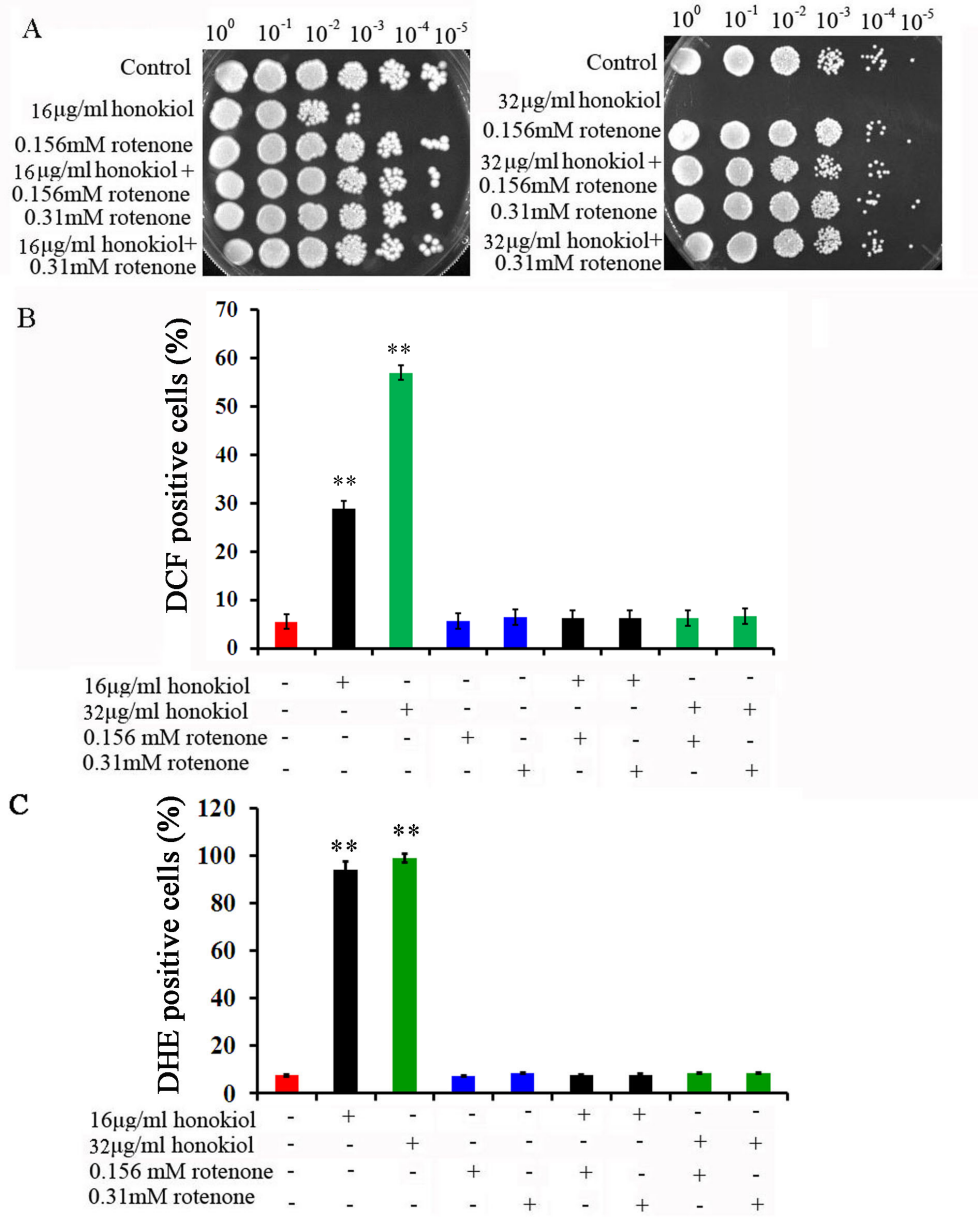
Effect of rotenone on *C*. *albicans* survival and ROS production after honokiol treatment. (A) Effect of rotenone on *C*. *albicans* survival after honokiol treatment. The SC5314 cells were treated with rotenone at concentrations of 0.156mM or 0.31mM for 1h and then exposed to honokiol for 24h. Effect of rotenone on H_2_O_2_ (B) and O_2_^•−^(C) production after honokiol treatment. ROS production was evaluated by flow cytometry as described in Materials and Methods. **p<0.01.

**Fig 3:**
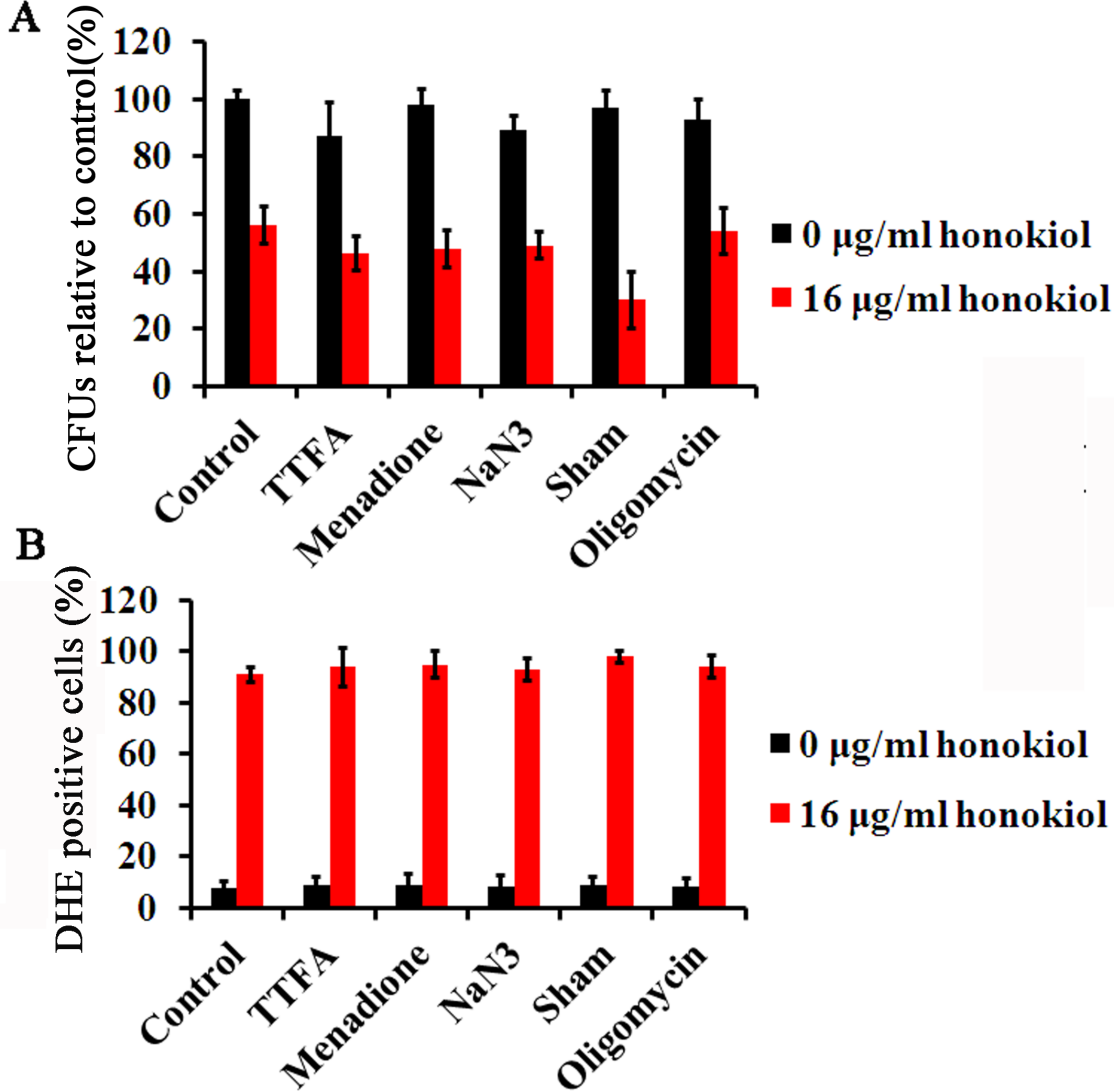
Effect of other mitochondrial respiratory chain inhibitors on *C*. *albicans* survival and ROS production after honokiol treatment. (A) Effect of respiratory chain inhibitors on *C*. *albicans* survival after honokiol treatment. The SC5314 cells were treated with respiratory chain inhibitors for 1h and then exposed to honokiol for 24h. (B) Effect of respiratory chain inhibitors on O_2_^•−^ production after honokiol treatment. O_2_^•−^ production was evaluated by flow cytometry as described in Materials and Methods. TTFA, thenoyltrifluoroacetone (0.125mM); menadione (0.0625mM); NaN_3_ (0.0025%); Sham, salicylhydroxamic acid (5mM); oligomycin (5μg/ml).

O_2_^•−^ typically arises because of electron leakage or reverse electron transport from the electron transport chain onto O_2_ during aerobic respiration [7]. To further investigate the sites of O_2_^•−^ production in mitochondria, we first used rotenone (respiratory chain C I inhibitor) to observe the effect on *C*. *albicans* killing and ROS production by honokiol. Pre-cultured with rotenone (0.156mM or 0.31mM) eliminated the antifungal activities of honokiol when the concentration was 16μg/mL (Fig 2A). We even tested the fungicide concentration 32μg/mL of honokiol when combined with two different concentrations of rotenone, yet the results were the same. In addition, ROS production (both H_2_O_2_ and O_2_^•−^) by honokiol was also completely eliminated by rotenone (Fig 2B and 2C). Rotenone can completely block both the antifungal activity of honokiol and ROS production (Fig 2). Then, we used several other respiratory chain inhibitors to observe the effect on *C*. *albicans* killing and ROS production by honokiol. Pre-cultured with thenoyltrifluoroacetone (TTFA), menadione, sodium azide (NaN3), and oligomycin showed almost no effect on honokiol-induced *Candida* killing and O_2_^•−^ accumulation (Fig 3). Salicylhydroxamic acid (Sham), a cyanide-resistant alternative oxidase inhibitor, could slightly enhance the susceptibility to honokiol and raised the level of O_2_^•−^ (Fig 3). These data indicated that the potential antifungal activity of honokiol was related to mitochondrial C I. Nevertheless, the mechanisms and consequences of such an interaction still require further investigation.

### Respiratory activity in *C*. *albicans* after exposure to honokiol

**Fig 4:**
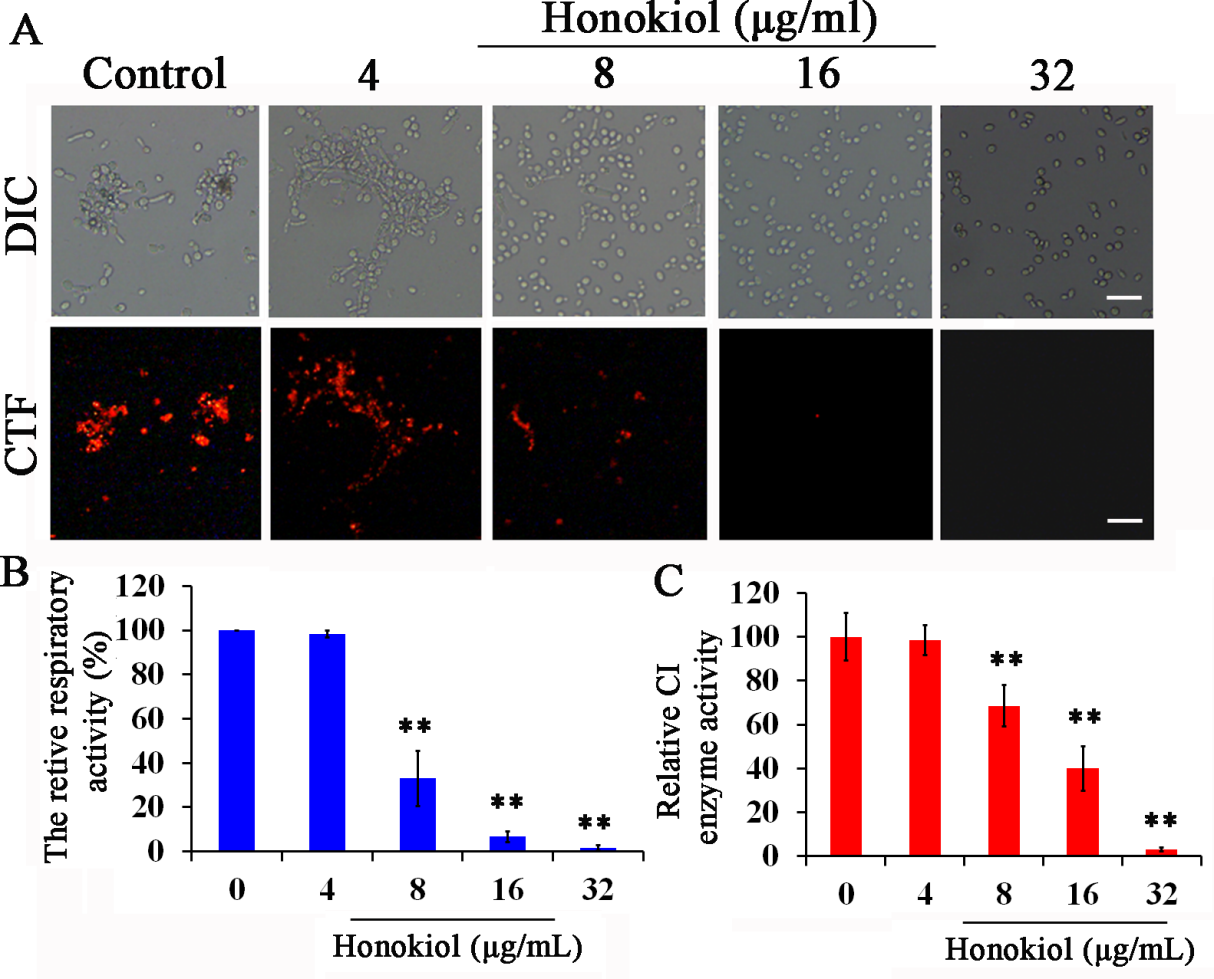
Effect of honokiol on respiratory activity and C I enzyme activity in *C*. *albicans*. (A) *C*. *albicans* SC5314 stained with CTC. (B) The relative fluorescence intensity was obtained by flow cytometric analysis of CTF-stained cells. (C) Effect of honokiol on C I enzyme activity. Scale bar = 10μm. **p<0.01.

CTC staining represents an index of the respiratory activity of the cell at the time of observation [19].CTC is a soluble crystal that forms a nearly colorless non-fluorescent solution. In the electron transport system, CTC, as an artificial redox partner, was reduced to a fluorescent formazan crystal (CTF) primarily by membrane-bound NADH-dehydrogenase [19]. The accumulation of CTF red fluorescence was clearly observed in control group, but honokiol induced a concentration-dependent decrease in respiratory activity (Fig 4A). Almost no CTF accumulation was observed both in 16μg/mL and 32μg/mL of honokiol-treated cells, indicating that the respiratory activity of *C*. *albicans* was blocked by honokiol. These results were also confirmed by flow cytometry (Fig 4B). We further investigated the C I enzyme activity in *C*. *albicans* after honokiol exposure. As shown in Fig 4C, the C I enzyme activities in *C*. *albicans* after honokiol treatment at 8μg/mL and 16μg/mL were reduced to 68.5% and 39.9%, respectively. In addition, 32μg/mL honokiol could almost completely inhibit the activity of C I enzyme. There was no significant difference between 4μg/mL honokiol treatment and the control group.

### The effect of honokiol on mtDNA in *C*. *albicans*

**Fig 5:**
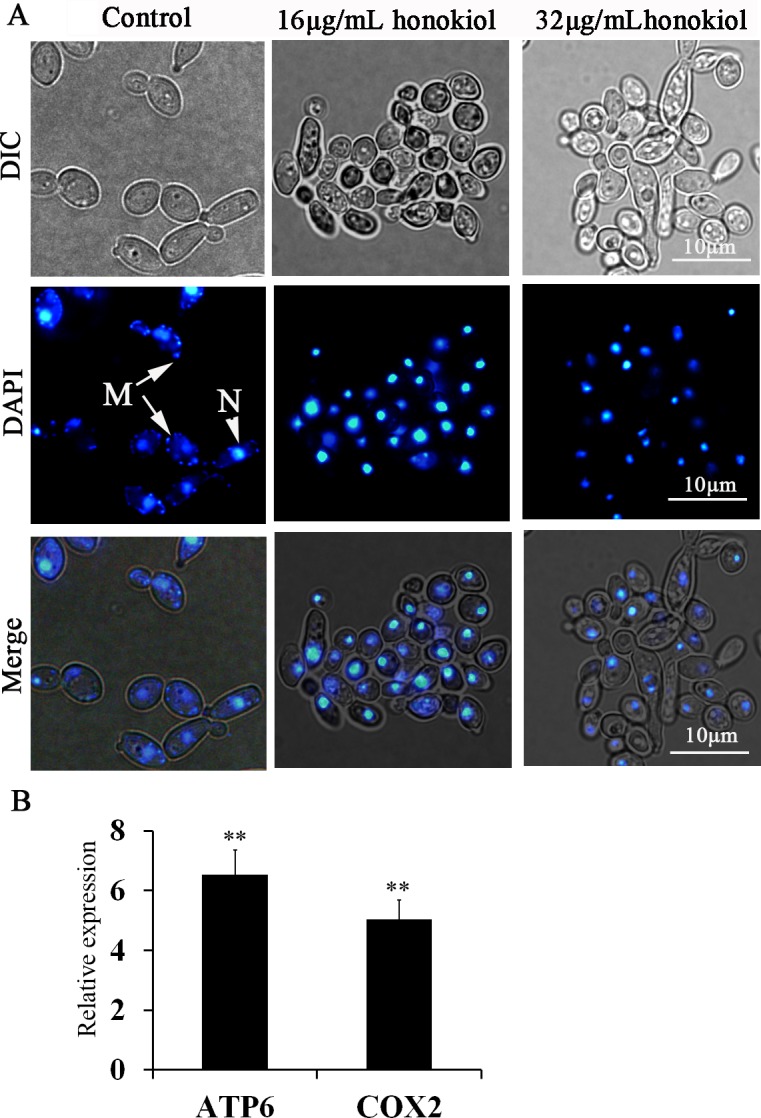
The effect of honokiol on mtDNA in *C*. *albicans*. (A) Nuclear and mtDNA was stained with DAPI. Nuclear DNA stained brightly, while the mitochondrial nucleoids displayed punctuate cytoplasmic staining. N, nucleus; M, mtDNA. (B) qRT-PCR analyzed the expression of mtDNA genes. Expression is relative to control group and the data are presented as the average of three biological replicates each normalized to ACT1. **p<0.01.

Staining with DAPI revealed that honokiol-treated cells were devoid of mtDNA, while the control group showed the punctuate staining for mtDNA when observed under fluorescence microscope (Fig 5A). In addition, we observed a significant induction of mitochondrial-encoded subunit 1 of cytochrome c oxidase (COX1) gene and subunit 6 of the F0 sector of mitochondrial F1F0 ATP synthase (ATP6) gene, compared with control (Fig 5B).

### Honokiol treatment increases the sensitivity to cell wall-perturbing agents

**Fig 6:**
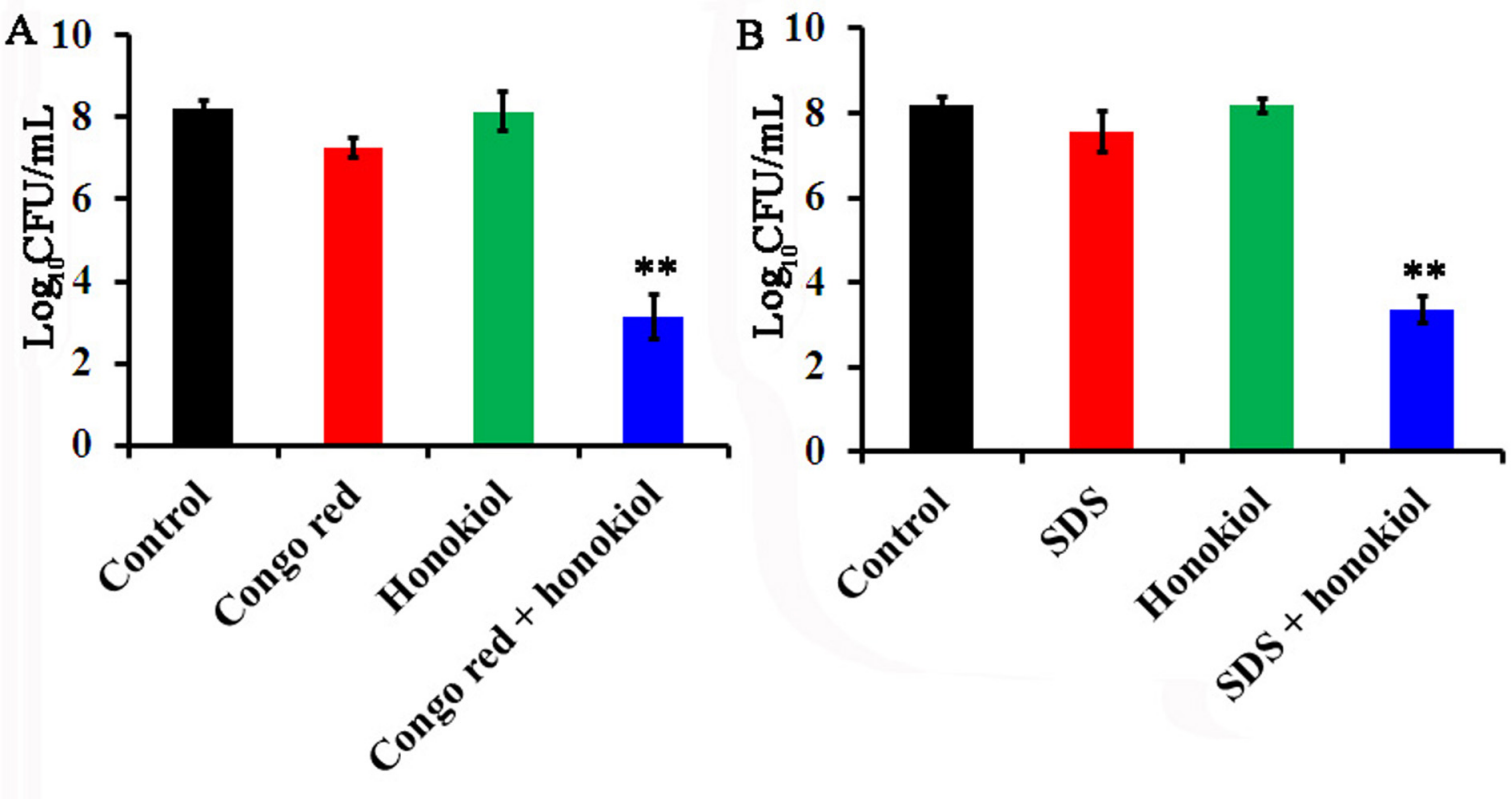
Honokiol treatment increases the sensitivity to cell wall-perturbing agents against *C*. *albicans*. (A) Honokiol treatment increases the sensitivity to Congo red. (B) Honokiol treatment increases the sensitivity to SDS. Cells were treated with honokiol (4μg/mL), cell-wall perturbing agents (Congo red, 4μg/mL; SDS, 0.01%) or their combination for 24 h. Viability detections were described in Materials and Methods. **p<0.01.

Mitochondrial dysfunction in *C*. *albicans* is reported to be linked with loss of cell wall integrity [27–29]. Therefore, we compared the sensitivities of *C*. *albicans* to the cell-wall perturbing agents Congo red (4μg/mL) or SDS (0.01%) alone or in combination with honokiol (4μg/mL). As shown in Fig 6, honokiol significantly increased the sensitivity of the cell wall-perturbing agents against *C*. *albicans*.

### Transcriptional analysis of honokiol-treated *C*. *albicans*

**Fig 7:**
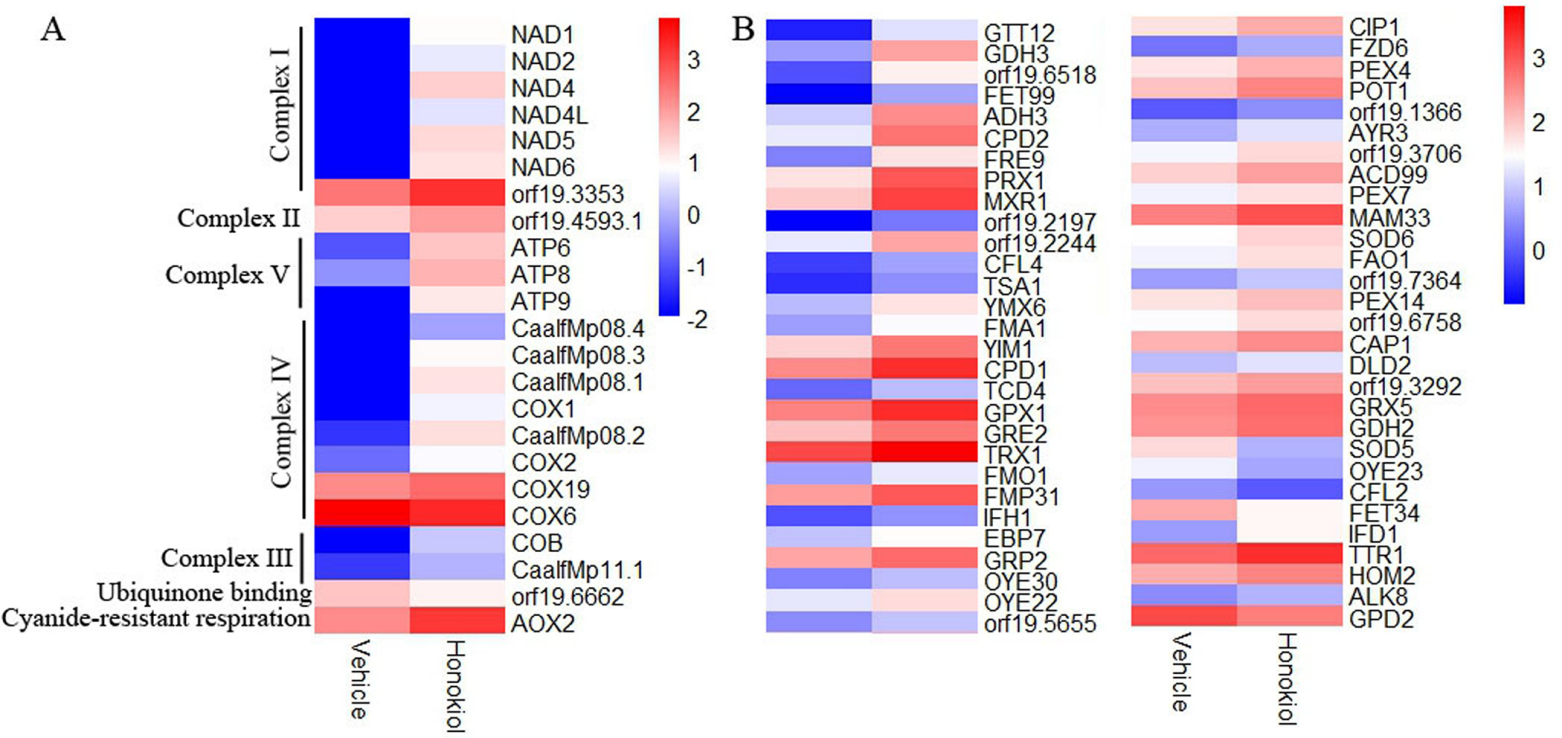
**Transcriptional profiling of mitochondrial electron transport (A) and oxidation-reduction process (B) in *C*. *albicans* SC5314 after honokiol treatment.** The expression level of genes which are represented by log_2_ (RPKM) values are indicated from blue to red.

**Fig 8:**
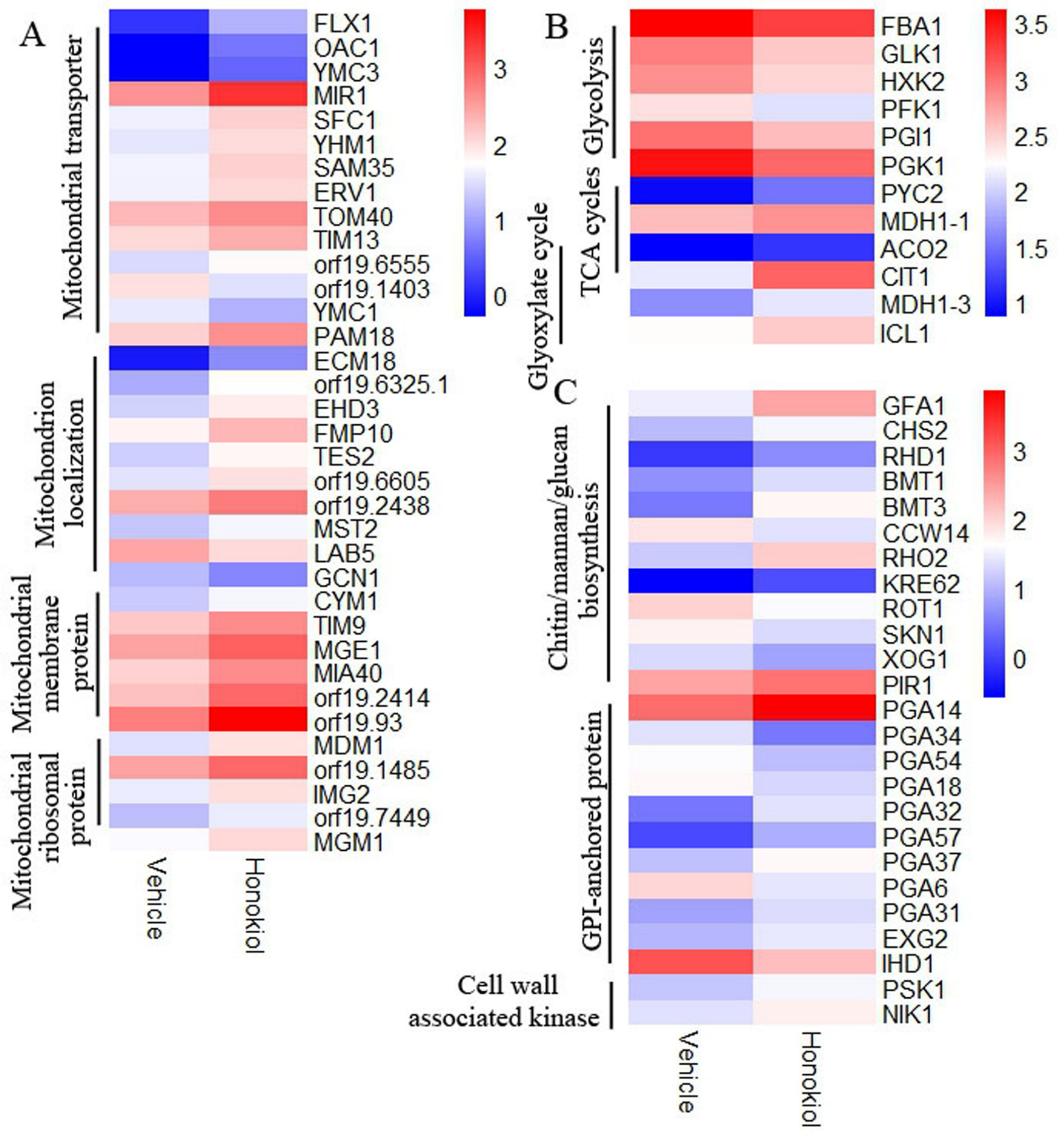
Transcriptional profiling of genes in response to honokiol treatment. Functional annotation for genes are shown: (A) genes of mitochondrial transporter, mitochondrion localization, mitochondrial membrane protein, and mitochondrial ribosomal protein. (B) genes involved in carbon metabolism, such as glycolysis, TAC cycles, and glyoxylate cycle. (C) genes involved in cell wall. The expression level of genes which are represented by log_2_ (RPKM) values are indicated from blue to red.

In order to find pathway and processed involved in generating superoxide of *C*. *albicans* treatment by honokiol, we performed a genome-wide transcriptome analysis of *C*. *albicans* SC5314 after honokiol treatment, 1152 genes were differentially expressed in vehicle-treated group vs. honokiol-treated group, of which 837 were significantly upregulated and 315 genes were significantly downregulated. RNA sequencing reveals 116 genes involved in mitochondrial and oxidation reduction were differentially expressed in honokiol-treated group (Fig 7). Because honokiol decreased mitochondrial respiratory chain C I enzyme activity and induced mitochondrial dysfunction, we focused on genes related to mitochondrial function. The genes encoding mitochondrial ETC proteins of CⅠ(NAD1, NAD2, NAD4, NAD4L, NAD5, NAD6, and orf19.3353), CⅡ (*orf19*.*4593*.*1*), CⅢ (COB and CaalfMp11.1), CⅣ (COX1, CaalfMp08.1, CaalfMp08.2, CaalfMp08.4, COX2, and COX19), CV (ATP9, ATP6, and ATP8), and alternative oxidase (AOX2, involved in a cyanide-resistant respiratory pathway) were upregulated after honokiol exposure (Fig 7A). Honokiol, having pro- and antioxidant activities, strongly induced the expression of genes involved in the oxidation–reduction process (Fig 7B). Besides the genes involved in mitochondrial electron transport and oxidation–reduction process, the mitochondrial transporter genes (TOM40, OAC1, FLX1, YMC3, SFC1, YHM1, SAM35, ERV1, TIM13, MIR1, PAM18, and orf19.6555), mitochondrial membrane (CYM1, TIM9, MGE1, MIA40, orf19.2414, and orf19.93) and ribosomal protein (MDM1, orf19.1485, IMG2, orf19.7449,and MGM1), and genes involved in mitochondrion localization (ECM18, orf19.6325.1, EHD3, FMP10, TES2, orf19.6605, orf19.2438, MST2, LAB5, and GCN1) are significantly upregulated (Fig 8A). In addition, the expression of genes involved in carbon metabolism, such as glycolysis, three carboxylic acid (TAC) cycles, and glyoxylate cycle were changed. Honokiol treatment downregulated the transcriptional levels of glycolysis (FBA1, GLK, HXK2, PFK1, PGI1, and PGK1) while upregulated three carboxylic acid cycles (ACO2, CIT1, and MDH1-1), and glyoxylate cycle genes (ACO2, CIT1, MDH1-3, and ICL1) (Fig 8B). We note that the transcription in several categories of cell wall protein (RBR2, SCW4, DSE1), cell wall chitin/hexosamine biosynthesis (GFA1, CHS2), phosphopeptidomannan, mannosyltransferase (RHD1, BMT1, BMT3), glucan synthase (RHO2, KRE62, PIR1), and GPI-anchored cell wall proteins (EXG2, PGA14, PGA31, PGA32, PGA57, and PGA37) were all upregulated expression (Fig 8C).

### GO assessment

Gene annotation and classification with GO terms for *C*. *albicans* was performed in *Candida* Database. The DEGs were mapped to GO ontologies of cellular components, molecular functions, and biological processes for GO enrichment analysis (Table 1). GO analysis revealed DEGs in honokiol treatment were enriched for 9 GO terms. The largest number of DEGs were enriched in oxidoreductase activity for GO molecular function (GO:0016491). Changes in biological processes included ribosome biogenesis (GO:0042254), electron transport and oxidative phosphorylation (GO:0042775, GO:0006119, GO:0042773, GO:0022904). In addition, the term enrichment for cellular component were respiratory chain (GO:0070469 and GO:0005746) and proteasome complex (GO:0000502).

**Table 1:** GO enrichment analysis.

Term		TermName			DEGs number	Gene name	Padj^a^
Ontology: Biological process							
GO:0042254			ribosome biogenesis	31		orf19.5991,RRS1,NOC4,orf19.6234,UTP21,DIM1,SPB4,YTM1,NOG1,KRR1,orf19.6828,IMP4,DBP8,RPS8A,HCA4,UBI3,orf19.1646,PES1,MRT4,NSA1,YST1,orf19.3393,NEP1,RCL1,TSR1,NOP5,RPL8B,RPL82,orf19.3778,GAR1,orf19.1388	0.00578
GO:0042775			mitochondrial ATP synthesis coupled electron transport	14		CaalfMp11.1,COX1,CaalfMp08.4,CaalfMp08.1,COB,NAD4L,NAD1,NAD4,CaalfMp08.2,COX2,NAD5,NAD2,NAD6,CaalfMp08.3	0.00604
GO:0006119			oxidative phosphorylation	14		NAD6,CaalfMp08.3,COX2,CaalfMp08.2,NAD5,NAD2,NAD4L,NAD1,NAD4,CaalfMp11.1,COX1,CaalfMp08.1,CaalfMp08.4,COB	0.01208
GO:0042773			ATP synthesis coupled electron transport	14		NAD5,NAD2,CaalfMp08.2,COX2,CaalfMp08.3,NAD6,CaalfMp08.4,CaalfMp08.1,COB,CaalfMp11.1,COX1,NAD4,NAD1,NAD4L	0.01208
GO:0022904			respiratory electron transport chain	14		CaalfMp08.3,NAD6,NAD5,NAD2,CaalfMp08.2,COX2,NAD4,NAD1,NAD4L,CaalfMp08.1,COB,CaalfMp08.4,CaalfMp11.1,COX1	0.04127
Ontology: Molecular function							
GO:0016491	oxidoreductase activity			111		XYL2,NAD4L,OYE22,orf19.3706,ADH4,FET99,DLD2,orf19.6518,orf19.7288,CaalfMp11.1,CaalfMp08.1,ADH3,PXP2,orf19.7306,EBP7,orf19.1366,SSP96,orf19.5565,orf19.2175,MXR1,IFM3,orf19.1639,NAD4,OYE23,orf19.2782,orf19.1438,orf19.5728,orf19.1340,orf19.2312,AOX2,YIM1,POX1,NAD2,GDH3,GPD2,SOD6,orf19.6066,IFD3,orf19.4567,spliced|Uncharacterized,orf19.2177,orf19.1844,orf19.86,FRE7,POX18,PRX1,NAD1,RNR1,orf19.1461,ERO1,RNR22,ERV1,TRX1,orf19.6341,NAD6,CFL2,orf19.1802,CFL4,GRP2,orf19.7204,FMO1,GRE2,FOX2,IFD6,ARG5,6,FET34,ADH2,orf19.4476,COB,HOM2,SOD5,orf19.5978,orf19.320,MIA40,orf19.7364,CaalfMp08.2,FRE9,orf19.6838,NAD5,TTR1,COX6,orf19.355,FRE30,orf19.5879,orf19.3537,orf19.1709,COX2,PUT2,ALK2,IFK2,orf19.6869,orf19.6306,COX1,CaalfMp08.4,CaalfMp08.3,orf19.3711,orf19.2394,MDH1-3,orf19.6398,GDH2,YMX6,orf19.2197,orf19.5655,TSA1,orf19.7495,orf19.2244,orf19.22.1,orf19.6143,orf19.6758,orf19.3292,ALK8	0.00949
Ontology: Cellular component							
GO:0005746	mitochondrial respiratory chain			14		CaalfMp08.3,NAD6,NAD5,NAD2,CaalfMp08.2,COX2,NAD4,NAD1,NAD4L,CaalfMp08.4,CaalfMp08.1,COB,CaalfMp11.1,COX1	0.00149
GO:0000502	proteasome complex			18		orf19.6973,orf19.213,RPT2,PUP2,orf19.1993,PRE8,RPN2,RPN3,PUP3,ECM29,RPN1,RPN10,PRE9,PRE10,orf19.2755,orf19.5961,CIC1,orf19.2301	0.00854
GO:0070469	respiratory chain			15		NAD1,NAD4L,NAD4,COX1,CaalfMp11.1,CaalfMp08.1,AOX2,CaalfMp08.4,COB,NAD6,CaalfMp08.3,CaalfMp08.2,COX2,NAD2,NAD5	0.0169

^a^Significantly enriched representative GO terms (adjusted p-value<0.05) are listed in table.

### KEGG analysis

**Fig 9:**
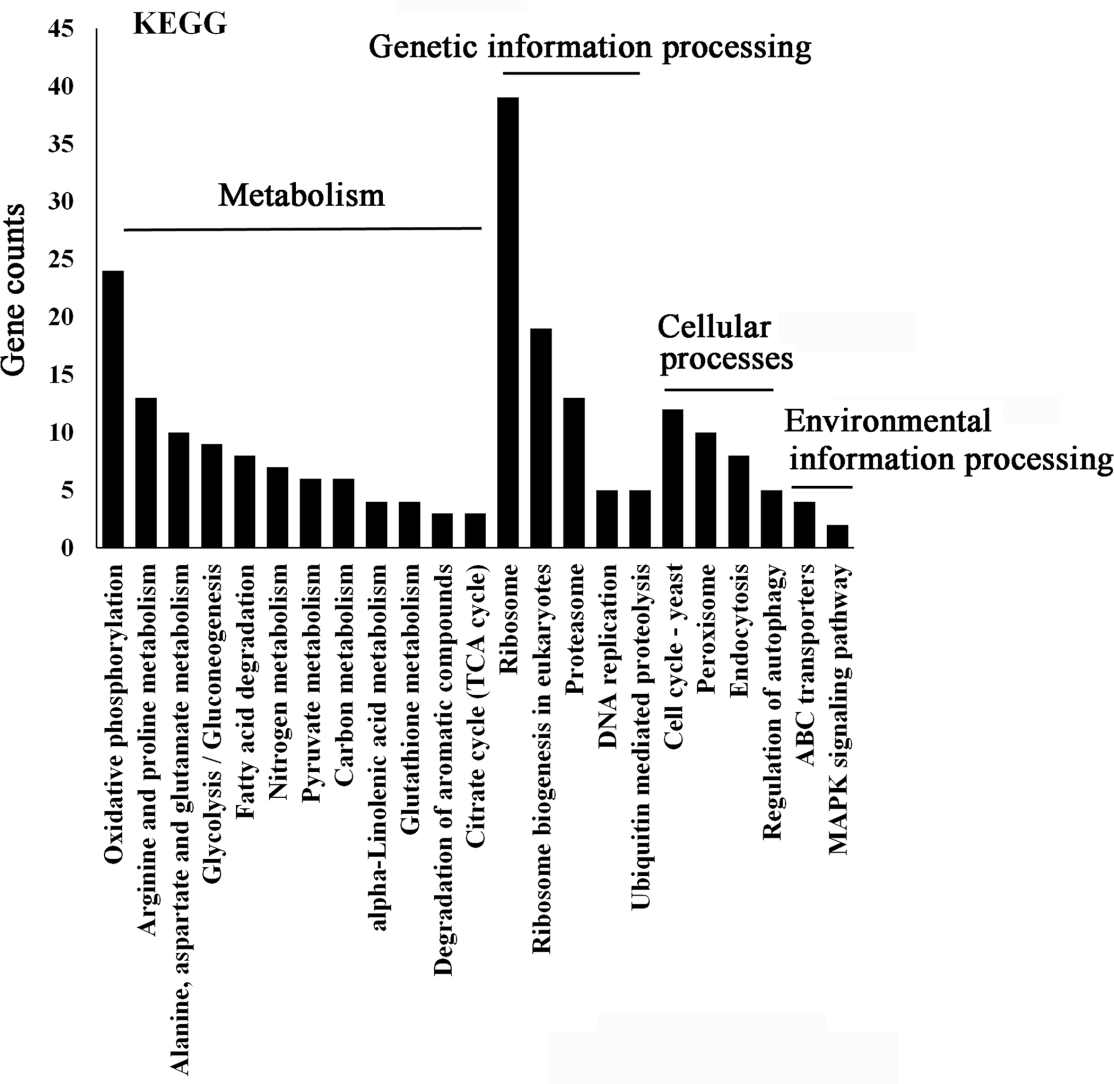
KEGG pathway annotation in response to honokiol treatment. The bottom x-axis indicates he main category. The y-axis indicates the number of genes in a specific category. The results are summarized in the following four main categories: (1) metabolism;(2) genetic information processing; (3) cellular precesses; (4) environmental information processing.

We also employed the KEGG pathway database to determine if the DEGs belong to particular signaling pathways. KEGG pathway mapping is the process to map molecular datasets, especially large-scale datasets, to the KEGG pathway maps for biological interpretation of higher-level systemic functions. The signaling pathway hypothesized to be affected by honokiol treatment mainly included signaling pathways related to metabolism (oxidative phosphorylation, arginine and proline metabolism, alanine, aspartate and glutamate metabolism,glycolysis/gluconeogenesis, fatty acid degradation, nitrogen metabolism, pyruvate metabolism, carbon metabolism, alpha-linolenic acid metabolism, glutathione metabolism, degradation of aromatic compounds, and citrate cycle), genetic information processing (ribosome, ribosome biogenesis in eukaryotes, proteasome, DNA replication, and ubiquitin mediated proteolysis), cellular processes (cell cycle, peroxisome, endocytosis, and regulation of autophagy), and environmental information processing (ABC transporters and MAPK signaling pathway) (Fig 9).

## Discussion

*Candida* species are a group of opportunistic fungal pathogens in humans, particularly among immunocompromised and hospitalized population [1–3]. Only few classes of antifungals such as azoles, flucytosine, polyenes, allylamines, and echinocandins are available for the treatment of *Candida* infections [30]. However, the rising drug resistance is an inevitable problem [31]. Therefore, it is necessary to discover new antifungal agents or other safer alternatives to improve the effectiveness of treatment of *Candida* infections [32,33]. Honokiol is an interesting small-molecule exhibiting pro- and antioxidant activities, similar with vitamin C and E [14]. Past studies have also demonstrated that honokiol has antifungal activities (MIC rang, 8–32 μg/mL) and when used in combination with fluconazole, can exhibit synergistic activity against clinical isolates of fluconazole-resistant *C*. *albicans* [20,34,35].To better understand how honokiol works on a cellular level, we sought to explore its antifungal mechanism and found honokiol caused an increase of intracellular ROS, lipid peroxidation, and protein oxidation in a dose-dependent manner in *C*. *albicans* [15]. This study will aim to clarify specific mitochondrial ROS-generation components that are involved in honokiol treatemt. Mitochondria have always been considered as a primary source of ROS like the superoxide anion radicals, which are converted from O_2_ by electron leakage from the mitochondrial transport chain during cellular respiration [6,7].

In the current study, we demonstrated that superoxide radicals was mainly generated and accumulated in mitochondria after honokiol exposure by using fluorescent ROS probes DHE and DCFH-DA (Fig 1). The mitochondrial respiratory chain is the major source of intracellular ROS generation. About 1–2% of the molecular oxygen consumed during normal physiological respiration is converted into superoxide radicals who serve as the precursor of most ROS [6]. There is growing evidence that most of the superoxide radicals generated by mitochondria is produced by C I and C Ⅲ[7,8]. To obtain an insight into intra-mitochondrial site of honokiol-induced overproduction of superoxide radicals, we used specific respiratory chain inhibitors. Only rotenone can completely block superoxide radicals generation and deprived of fungicidal activity of honokiol (Figs 2 and 3). Since rotenone is a C I inhibitor, we hypothesis that honokiol may compete with rotenone at or close to the quinone binding sites of C I or the binding site of honokiol is upstream from the rotenone binding site. In addition, we also found mitochondrial NADH dehydrogenase activity was decreased after honokiol, indicating the C I activities were inhibited (Fig 4). Consistent with the above results, respiratory activity was decreased, which may be associated with the inhibition of C I (Fig 4). In view of the fact that O_2_^•−^ production is completely inhibited by the addition of a specific C I ubiquinone–site inhibitor rotenone that prevents C I binding to CoQ, we concluded the mechanism by which honokiol produces large amounts of O_2_^•−^ is specifically through respiratory C I reverse electron transport [36,37].

One important target of ROS is the mitochondrial DNA due to the close proximity to the electron transport chain, the major locus for free radical production, and the lack of protective histones [38]. DAPI staining revealed that honokiol treatment caused loss of mtDNA, especially after 32μg/mL honokiol treatment, the cells were completely devoid of mtDNA. However, we observed increased transcription of mitochondrial genes (ATP6 and COX2) after honokiol exposure (Fig 5). It is possible that cells after honokiol exposure attempts to overcome the ROS-induced mtDNA damage by increased mtDNA genes expression, which reflects a compensatory mechanism [39,40].

We identified a number of DEGs from honokiol treatment. After honokiol treatment, we observed increased expression of genes involved in mitochondrial respiratory electron transport chain, oxidoreductase activity, and ribosome biogenesis (Fig 7 and Table 1). C I is the first step in the respiratory chain and is located in the inner mitochondrial membrane [7]. The molecular mass of CI is around 800kDa and is composed of at least 39 subunit proteins [29]. To date, the CI structure in *C*. *albicans* remains somewhat unclear. *C*. *albicans* has 14 core CI genes of bacterial progenitor that are proton-pumping NADH: ubiquinone oxidoreductases, seven of which are encoded by mitochondrial DNA (NAD1, NAD2, NAD3, NAD4, NAD4L, NAD5, and NAD6), the other subunit genes are nuclear-encoded. Since methods to selectively mutate the mitochondrial genome in *C*. *albicans* are unavailable, the core CI genes encoded by mitochondrial DNA in *C*. *albicans* are not studied yet [29]. Our further work is required to clarify the interaction of honokiol with mitochondrial C I.

**Fig 10:**
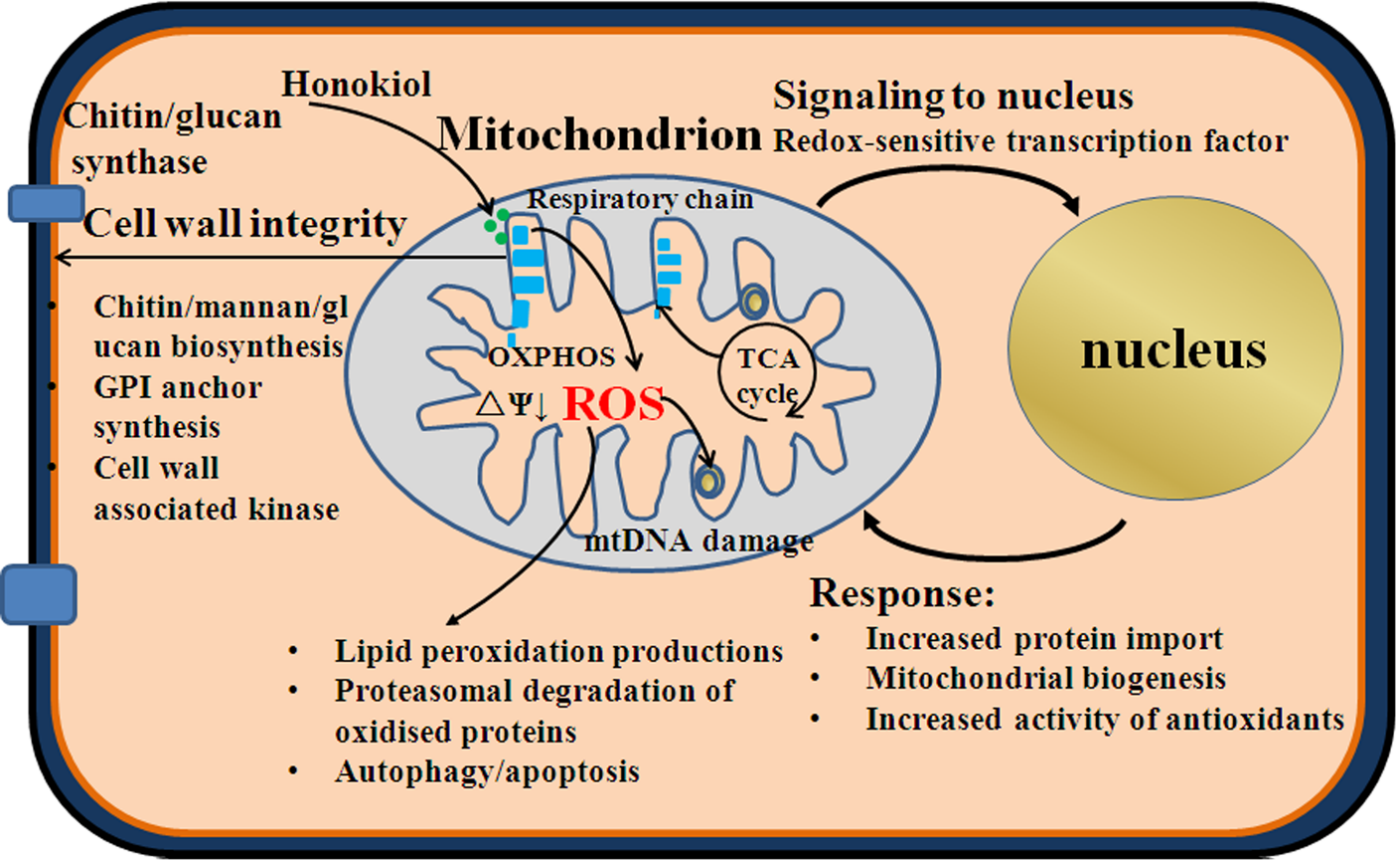
A model of the cellular response to honokiol treatment. Honokiol targets on the mitochondrial respiratory electron transport chain C I inducing ROS production (mainly O_2_^·−^) and mitochondria dysfunction. ROS induces mtDNA damage, mtΔψ decrease, oxidation of proteins and lipids. The transcriptome indicates that the cellular stress response increases including increased protein import to mitochondria, upregulate the expression of antioxidant enzyme genes, remove oxidized proteins by the ubiquitin-dependent proteasome system. Mitochondrial dysfunction in *C*. *albicans* is associated with loss of cell wall integrity. (1) affect the activity of wall synthesis enzymes in plasma membrane; (2) impinge on GPI anchor synthesis; (3)activation of cell wall integrity pathway upon cell wall stress. mtΔψ: mitochondrial membrane potential.

There are many lines of evidence to suggest that oxidative stress is associated with the action of honokiol [14,15]. Honokiol could trigger a multifaceted response (Fig 10). It activates stress response pathway to tolerate ROS toxicity, including increased expression of genes involved in the oxidative stress response (CAP1, *GTT12*, *PRX1*, *MXR1*, *GPX1*, *TRX1*, *IFH1*, and *SOD6*), proteolysis and protein degradation (*UBC4*, *UBC15*, *UBC8*, and *UBP6*), and autophagy (*ATG8*, *ATG9*, *ATG27*, *orf19*.*3998*, *APG13*, and *PRB1*) (Fig 8 and S2 Table). CAP1, an AP-1 bZIP transcription factor, was significantly upregulated after honokiol treatment. Cap1 regulates a variety of oxidative stress response including the cellular antioxidant defense system, carbohydrate metabolism and energy metabolism, protein degradation, ATP-dependent RNA helicase, and resistance pathways [41]. Redox homeostasis in cells is important for maintaining proper cellular functions [42]. Honokiol targets on mitochondrial respiratory chain C I, inducing ROS accumulation, disruption of intracellular redox homeostasis, irreversible oxidative modifications of lipid, protein or DNA, and activation of autophagy or apoptosis signaling pathway.

There are many lines of evidence to suggest that mitochondrial dysfunction in *C*. *albicans* is known to be linked with loss of cell wall integrity [27–29]. The fungal cell wall has vital roles in growth, survival, morphogenesis, and pathogenicity. Critical for the coordination of these activities is the dynamic nature of the cell wall, its ability to respond to external and internal stimuli [43]. Altered sensitivities to cell wall perturbing agents indicate modified cell wall properties [43,44]. In our study, honokiol treatment resulted in an increased sensitivity to Congo red and SDS (Fig 6). In addition, the genes for cell wall synthesis have three major changes in transcriptional levels (Fig 8): 1). chitin/mannan/glucan biosynthesis, such as upregulated CHS2 which encodes chitin synthase and BMT3 which encodes β-mannosyltransferase is required for β-mannan elongation either in phosphopeptidomannan or in phospholipomannan [44]. Reduced glucanase gene expression, for example, SKN1 encoding a N-glycosylated type II membrane protein with a predicted role in β-1,6-glucan synthesis was downregulated after honokiol exposure; 2). GPI anchor protein, such as PGA31, EXG2, PGA32, PGA57, and PGA37 were upregulated. GPI-anchored proteins are important cell surface proteins in *C*.*albicans*, and they have important impact on the adhesion,morphogenesis and cell wall synthesis [44]; 3). cell wall associated kinase. such as PSK1(PAS kinase), a putative serine/threonine protein kinase, was upregulated after honokiol exposure. It has been reported that inhibition of cell wall biogenesis by caspofungin also causes an increase in PSK1 expression [44]. Honokiol treatment caused mitochondrial dysfunction and then damaged the cell wall integrity, thus, increased sensitivity to cell wall perturbing agents.

The present work shows that honokiol may target mitochondrial respiratory chain C I, leading to mitochondrial dysfunction, accompanied by increased cellular superoxide anion and oxidative stress (Fig 10). Use of antifungal agents that are mitochondrial respiratory chain C I inhibitors can also be toxic to mammalian cells associated with aging and age-related diseases such as Parkinson’s disease [45]. However, potential side effects of mitochondrial respiratory chain inhibitors as antifungal agents can be reduced if effective dosage can be diminished. Such a reduction can be achieved by use of honokiol as a chemosensitizer to azoles agents or echinocandins. This work not only provides insights on the mechanism by which honokiol interferes with fungal cell, demonstrating previously unknown effects on mitochondrial physiology, but also raises a note of caution on the use of *M*. *officinalis* as a Chinese medicine due to the toxic for mitochondria and suggests the possibility of using honokiol as chemosensitizer.

## Supporting information

S1 Table Gene-specific primers used for real-time RT-PCR. (DOC)

S2 Table Differential expression genes involved in the ribosome biogenesis, proteolysis and protein degradation, and autophagy. (XLSX)
