# Supplementary material for: Honokiol induces superoxide production by targeting mitochondrial respiratory chain complex I in Candida albicans
Source: PLoS One. 2017 Aug 30;12(8):e0184003. doi: 10.1371/journal.pone.0184003 (PMC5576747; doi:10.1371/journal.pone.0184003)
Supplement: S1 Table — (DOC) [file pone.0184003.s001.doc]

S1Table. Gene-specific primers used for real-time RT-PCR.

| Genes | Forward primer sequence (5’→3’) | Reverse primer sequence (5’→3’) |
| --- | --- | --- |
| *ACT1* | TTTCATCTTCTGTATCAGAGGAACTTATTT | ATGGGATGAATCATCAAACAAGAG |
| *ATP6* | AGGGAAGAAATGACCTCCAGC | CGTTTAGGTTCTACACGTTGAGG |
| *COX2* | AGATGCTACTCCAGGTCGTCT | CATACCGTGGTTAACCCCACA |
